# *Schizosaccharomyces pombe* Grx4, Fep1, and Php4: *In silico* analysis and expression response to different iron concentrations

**DOI:** 10.3389/fgene.2022.1069068

**Published:** 2022-12-07

**Authors:** Alia Ebrahim, Manal A. Alfwuaires, Mohammad H. Abukhalil, Fawaz Alasmari, Fawad Ahmad, Rui Yao, Ying Luo, Ying Huang

**Affiliations:** ^1^ Jiangsu Key Laboratory for Microbes and Genomics, School of Life Sciences, Nanjing Normal University, Nanjing, China; ^2^ Department of Biological Sciences, Faculty of Science, King Faisal University, Al-Ahsa, Saudi Arabia; ^3^ Department of Medical Analysis, Princess Aisha Bint Al-Hussein College of Nursing and Health Sciences, Al-Hussein Bin Talal University, Ma’an, Jordan; ^4^ Department of Biology, College of Science, Al-Hussein Bin Talal University, Ma’an, Jordan; ^5^ Department of Pharmacology and Toxicology, College of Pharmacy, King Saud University, Riyadh, Saudi Arabia

**Keywords:** iron homeostasis, *Schizosaccharomyces pombe*, Grx4, Fep1, Php4

## Abstract

Due to iron’s essential role in cellular metabolism, most organisms must maintain their homeostasis. In this regard, the fission yeast *Schizosaccharomyces pombe* (sp) uses two transcription factors to regulate intracellular iron levels: spFep1 under iron-rich conditions and spPhp4 under iron-deficient conditions, which are controlled by spGrx4. However, bioinformatics analysis to understand the role of the spGrx4/spFep1/spPhp4 axis in maintaining iron homeostasis in *S. pombe* is still lacking. Our study aimed to perform bioinformatics analysis on *S. pombe* proteins and their sequence homologs in *Aspergillus flavus* (af), *Saccharomyces cerevisiae* (sc), and *Homo sapiens* (hs) to understand the role of spGrx4, spFep1, and spPhp4 in maintaining iron homeostasis. The three genes’ expression patterns were also examined at various iron concentrations. A multiple sequence alignment analysis of spGrx4 and its sequence homologs revealed a conserved cysteine residue in each PF00085 domain. Blast results showed that hsGLRX3 is most similar to spGrx4. In addition, spFep1 is most closely related in sequence to scDal80, whereas scHap4 is most similar to spFep1. We also found two highly conserved motifs in spFep1 and its sequence homologs that are significant for iron transport systems because they contain residues involved in iron homeostasis. The scHap4 is most similar to spPhp4. Using STRING to analyze protein-protein interactions, we found that spGrx4 interacts strongly with spPhp4 and spFep1. Furthermore, spGrx4, spPhp4, and spFep1 interact with spPhp2, spPhp3, and spPhp5, indicating that the three proteins play cooperative roles in iron homeostasis. At the highest level of Fe, *spgrx4* had the highest expression, followed by *spfep1*, while *spphp4* had the lowest expression; a contrast occurred at the lowest level of Fe, where *spgrx4* expression remained constant. Our findings support the notion that organisms develop diverse strategies to maintain iron homeostasis.

## 1 Introduction

Due to iron’s critical role in cell metabolism, maintaining its balance is essential for most organisms (Maio et al., 2022). Low-intracellular iron concentration inhibits the activity of Fe-dependent enzymes, while high iron accumulation stimulates reactive oxygen species (ROS) formation and disrupts other metal-trafficking pathways (Vachon et al., 2012; Chen et al., 2022). Therefore, cells must tightly regulate iron uptake to maintain an equilibrium between adequate and excessive concentrations (Liu et al., 2022). In this regard, the fission yeast *Schizosaccharomyces pombe* (sp) uses two transcription factors, spFep1 and spPhp4, to regulate iron levels within cells (Mercier et al., 2006; Jbel et al., 2009). spFep1 is activated when iron is abundant (Jbel et al., 2009), whereas spPhp4 is activated when iron is scarce (Mercier et al., 2006; Attarian et al., 2018; Misslinger et al., 2021). spFep1 and spPhp4 collaborate to regulate each other’s transcriptomic expression levels in an iron-dependent manner (Vachon et al., 2012; Berndt et al., 2021).

When the amount of intracellular iron is high, spFep1 binds to chromatin, preventing the expression of several genes involved in iron acquisition (Pelletier et al., 2002; Labbé et al., 2007; Mao and Chen, 2019; Robinson et al., 2021). However, iron transporter gene transcription at low iron concentrations is activated due to spFep1’s inability to bind chromatin (Jbel et al., 2009; Berndt et al., 2021). Several fungi possess iron-dependent regulators with similar sequences and functions to spFep1, but not *S. pombe* (Chao et al., 2008; Haas et al., 2008; Hwang et al., 2012; Alkafeef et al., 2020). It was found that spFep1 interacts with monothiol glutaredoxin spGrx4 (Vachon et al., 2012; Miele et al., 2021). For instance, spGrx4 is essential for inhibiting spFep1 function when cells transition from iron-rich to iron-poor conditions (Jbel et al., 2011; Kim et al., 2011; Daniel et al., 2020). spGrx4’s glutaredoxin (GRX) domain forms a connection with spFep1 *via* its N-terminal (Gupta and Outten, 2020; Talib and Outten, 2021). The spFep1 DNA-binding domain is inactivated by the spFep1-GRX-binding domain, which in turn prevents the spFep1 DNA-binding domain from binding to chromatin, thereby inhibiting the expression of the gene of interest (Miele et al., 2021).

*spphp4* encodes a subunit of the CCAAT-binding protein complex that plays a critical role in regulating iron homeostasis (Mühlenhoff et al., 2020). This complex also contains three additional subunits, spPhp2, spPhp3, and spPhp5, which are highly expressed (Jbel et al., 2009; Jbel et al., 2011; Ohtsuka et al., 2021; Protacio et al., 2022). However, *spphp4* transcript levels are increased in iron-deficient conditions and decreased in iron-rich environments (Jbel et al., 2009). Although spPhp4 is not required for the complex’s DNA-binding activity, it is responsible for the Php complex’s transcriptional repression ability (Brault and Labbé, 2020). Furthermore, researchers used genome-wide microarray analysis and iron-starvation conditions and discovered that spPhp4 can coordinate the suppression of 86 genes (Jung et al., 2010; Martínez-Pastor and Puig, 2020). Many of these genes produce proteins involved in iron-dependent metabolic processes, such as heme biosynthesis, sulfur-iron cluster assembly, the tricarboxylic acid pathway, and mitochondrial respiration. Under iron-deficient conditions, DNA microarray analysis also revealed that the gene encoding the iron-responsive transcriptional inhibitor spFep1 exhibited spPhp4-dependentdown-regulation (Martínez-Pastor and Puig, 2020).

To our knowledge, bioinformatics analysis to understand the role of spGrx4, spFep1, and spPhp4 in maintaining iron homeostasis is still elusive. In this study, we conducted a bioinformatics analysis on *S. pombe* and their sequence homologs in *Aspergillus flavus* (af), *Saccharomyces cerevisiae* (sc), and *Homo sapiens* (hs) to understand the role of spGrx4, spFep1, and spPhp4 in maintaining iron homeostasis. Under various ferrous concentrations, expression analysis was also conducted to gain a deeper understanding of the critical roles of spGrx4, spPhp4, and spFep1 in iron homeostasis. In light of this, we proposed a model in which spFep1 and spPhp4 interact to regulate the expression of each other, thus controlling the strict regulation of intracellular iron concentrations (Jung et al., 2010). Our analyses of spGrx4 revealed a conserved cysteine residue in each PF00085 domain, and that human hsGLRX3 is most similar to spGrx4. spFep1 is highly correlated with scDal80. The scHap4 is most similar to spPhp4. Our analysis also showed that Grx4 interacts strongly with spPhp4 and spFep1, and that spGrx4, spPhp4, and spFep1 interact with spPhp2, spPhp3, and spPhp5, indicating that our three proteins play cooperative roles in iron homeostasis. At the highest level of Fe, *spgrx4* had the highest expression, followed by *spfep1*, while *spphp4* had the lowest expression; a contrast occurred at the lowest level of Fe, where *spgrx4* expression remained constant.

## 2 Materials and methods

### 2.1 Proteins data set and sequence analyses

The protein sequences of spGrx4, spPhp4, and spFep1 sequences were used as queries in BLAST searches against fungal and human genome databases, such as the NCBI, OrthoFinder and PHYLome, to identify sequence homologs. The E-value threshold of 0.05 was used to retrieve all protein sequences. We used TeUniProt Knowledgebase (UniProtKB) to determine whether the candidate proteins contained the conserved active site. Using SMART, Simple Modular Analysis Research Tool, the presence of conserved domains in Te candidate proteins was determined (Letunic and Bork, 2018). We utilized the Multiple EM for Motif Elicitation (MEME Suite) Version 5.4.1 (Peng et al., 2018) with the default settings to identify conserved sequence motifs (El-Sappah et al., 2021a; El-Sappah et al., 2021b).

### 2.2 Phylogenetic analysis

Multiple amino acid sequences were aligned to construct a phylogenetic tree using the MUSCLE algorithm incorporated into MEGA X software; version 10.0.5 (Kumar et al., 2018; Li et al., 2022). The phylogenetic tree was constructed using maximum likelihood (Jones-Taylor-Thornton model, 100 bootstrap replications) (Li et al., 2021).

### 2.3 Protein–protein interactions, prediction, and protein modeling

According to Kelley et al. (2015), the Phyre2 online webserver (sbg.bio.ic.ac.uk/phyre2/) was used for protein prediction, modeling, and analysis in the intensive mode. The STRING database (https://string-db.org) was used to analyze protein-protein interactions using the amino acid sequences.

### 2.4 Growth conditions and ferroptosis treatments

*S. pombe* cells were cultured overnight in YES medium at 30°C before diluting to an OD600 of 0.2 and re-culturing for the same period. The cells were then assembled and assigned an OD_600_value of 3.0. A tenfold dilution series was prepared, and each dilution was spotted on plates containing 3 μl YES media containing 0.25, 0.5, 3, or 6.0 mM Fe_2_ (SO_4_)_3_, or was left untreated.

### 2.5 RNA isolation and real-time quantitative PCR

Following the manufacturer’s instructions, total RNA was isolated from *S. pombe* cells using the High Pure RNA Isolation kit (Roche). Next, quantitative RT-PCR was conducted (Stratagene) using a FastStart SYBR Green Master kit (FSSGM; Roche) and the Mx3005P system. Four specific primers were employed for the following genes: *act1*, *grx4*, *fep1*, and *php4*, as follows (*act1*: Forward (F) 5′-ACT​ATG​TAT​CCC​GGT​ATT​GCC-3′, Reverse (R) 5′-GAC​AGA​GTA​TTT​ACG​CTC​AGG-3′; *grx4*: (F) 5′-GCG​TCG​CCT​ATT​GTG​CAA​GG-3′, (R) 5′-GTT​GGT​TGT​CGG​CAG​GTT​CG-3′; *fep1*: (F) 5′-CCC​GAA​GAG​CCA​CCC​TCA​AA-3′, (R) 5′-CCG​GCG​AGG​TAG​AGG​ATT​GG-3′; *php4*: (F) 5′-CAA​GAC​CTG​GGC​CGA​CTT​CA-3′, (R) 5′-AGC​CTG​CAG​CAT​CTC​CAA​CT-3′). We constructed the first-strand cDNA template in accordance with the manufacturer’s guidelines. Next, 50 µl of ultra-pure nuclease-free H_2_O was added to a mixture of 25 ml of FSSGM, 0.2 µM of each F and R primer, and 0.1 µg of cDNA. PCR was configured as follows: Pre-incubation at 95°C for 10 min, then 40 cycles of 95°C for 10 s, 55°C for 10 s, and 72°C for 20 s. The final step consisted of gradually increasing the temperature from 55°C to 95°C at a rate of 1°C/10 s to collect melting curve data (Islam et al., 2022). The reference (actin gene, act1) and target genes were serially diluted (1:10, 1:100), along with a non-template control, and added to each assay. Using melting curve analysis, we validated the specificity of each amplification reaction. In addition, we adjusted the expression levels for the reference gene act1, and Pfaffle (2001) calculated the relative gene expression levels using Pfaffle’s (2001) method.

## 3 Results

### 3.1 spGrx4, spFep1, and spPhp4 alignment and domain analysis

#### 3.1.1 spGrx4

**FIGURE 1:**
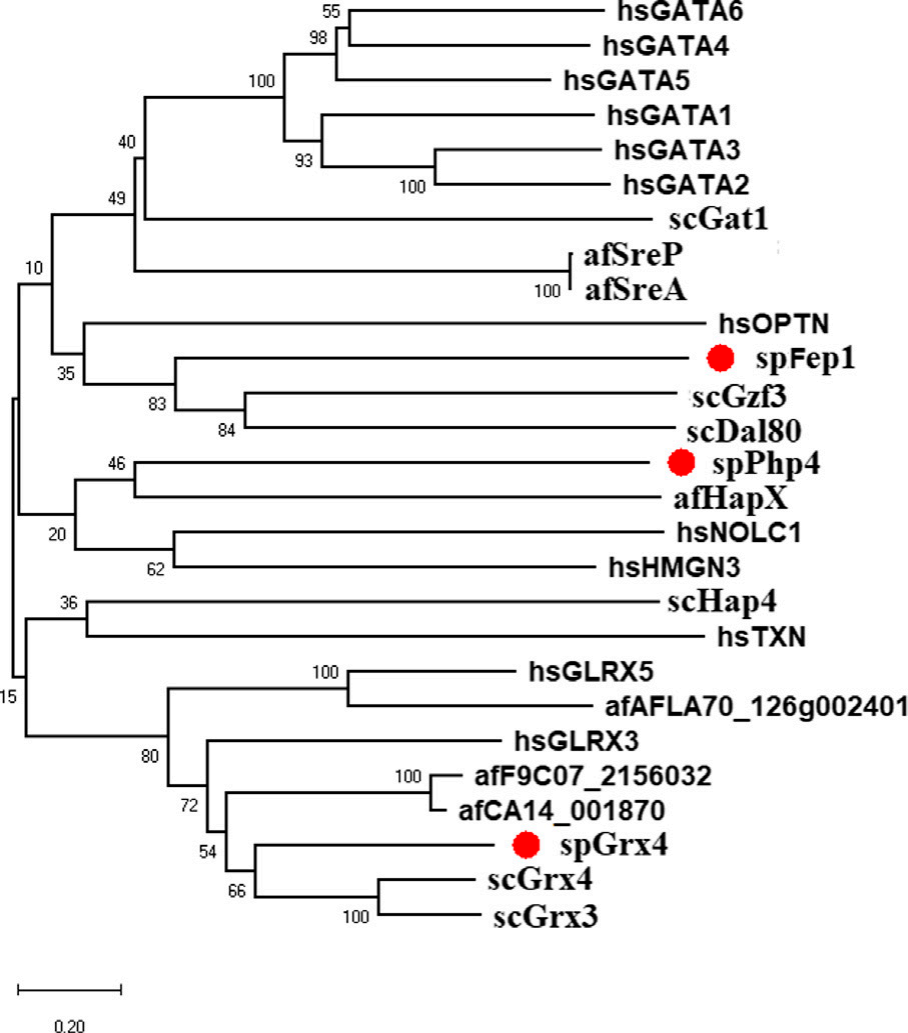
Bayesian evolutionary tree of spGrx4, spFep1, and spPhp4 and their sequence homologs in *A. flavus, S. cerevisiae*, and humans. spGrx4, spFep1, and spPhp4 are colored red. In the analysis, the strict clock model was implemented. Using the MCMC algorithm, the trustworthiness of phylogeny nodes was assessed. At the nodes, Bayesian posterior probabilities are displayed. The scale bar indicates that there are 0.2 amino acid substitutions at each site.

**FIGURE 2:**
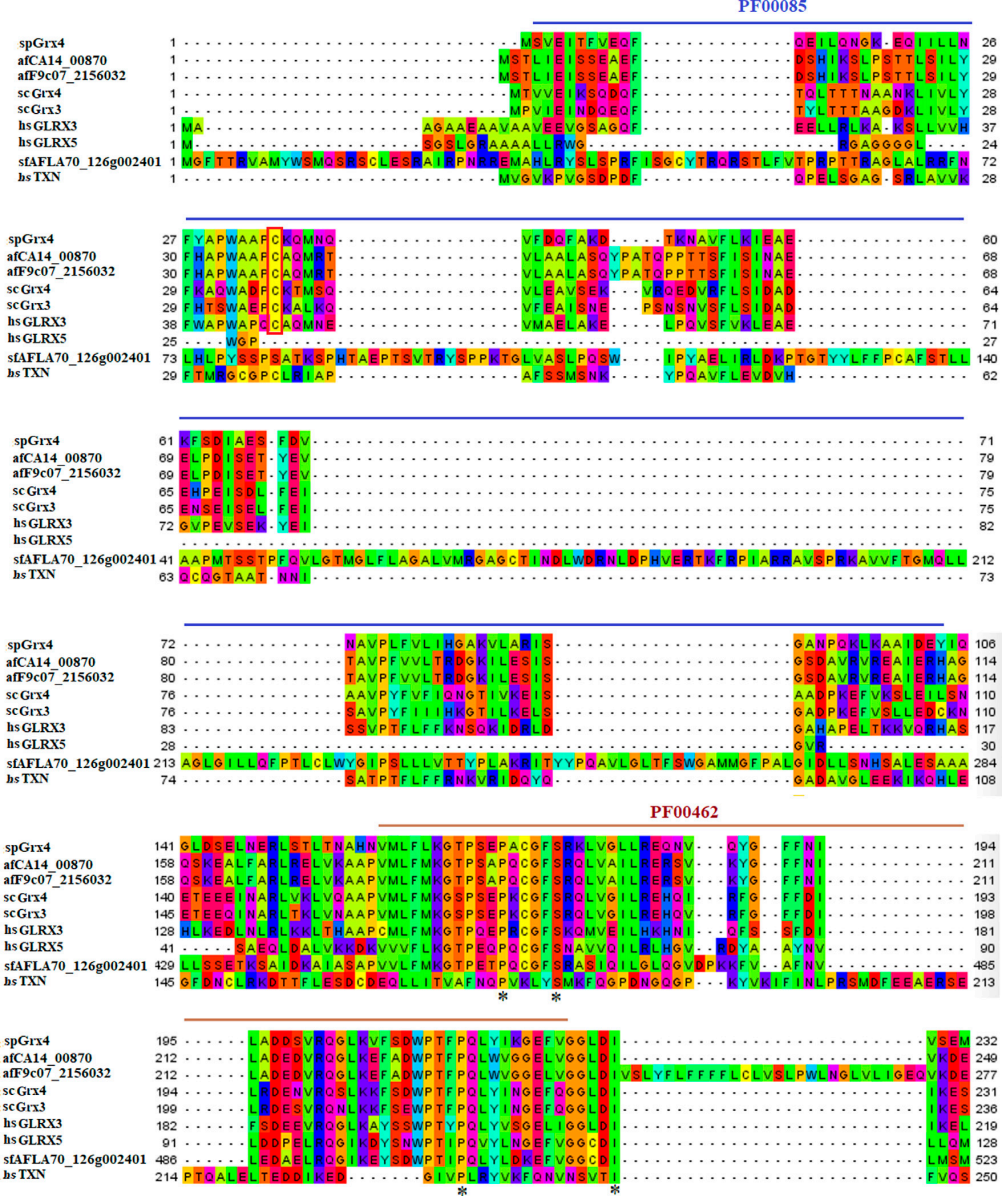
Multiple sequence alignment of the PF00085 and PF00462 domain sequences in spGrxp4 and its sequence homologs in *A. flavus, S. cerevisiae*, and humans using the MEGAX10.1.8 software. The Grx4 domains were discovered through a search of the SMART database. The two characteristic residues (a Cys residue in the PF00085 domain and a Cys172 residue in the PF00462 domain) are enclosed in square brackets. They are highlighted according to the biochemical properties of the amino acid residues.

As part of our study, we used the protein sequences of spGrx4, spFep1, and spPhp4 as query sequences, and performed BLAST searches against the human and fungal genome databases to determine the functional and structural diversity of these proteins. For further examination of spGrx4, eight spGrx4 homologs were chosen: two from *S. cerevisiae* (scGrx4 and scGrx3), three from *A. flavus* (afCA14001870, af F9C072156032, and afAFLA70126g002401), and three from humans. We found that hsGLRX5 was the most closely related by sequence homology to spGrx4 with a 50% identification rate (Table 1), while hsGLRX3 was predicted to be the functional homolog of spGrx4 based on the two major ortholog predictor tools (OrthoFinder and PHYLome). Using the maximum-likelihood method, we constructed a phylogenetic tree of Grx4 based on their similarity to estimate their evolutionary relationships (Figure 1). Our research demonstrates that spGrx4 is strongly associated with scGrx4. We also aligned the sequences of spGrx4 and its sequence homologs in humans, *A. flavus*, and *S. cerevisiae* to comprehend the functional and structural diversity of the proteins (Figure 2). Our multiple sequence alignment revealed that all selected Grx4 PF00085 domains contain a conserved cysteine (Cys) residue (Cys in spGrx4, afCA14001870, af F9C072156032, scGrx4, scGrx3, hsGLRX3 and hsGLRX5, and selenocysteine (Sec) in hsGPX1, hsGPX4, and hsGPX6). Moreover, our analysis revealed the presence of Cys^172^ in Grx4 PF00462 (All of selected proteins except in hsTXN).

**TABLE 1:** Sequence comparison of spGrx4 to its sequence homologs in *A. flavus*, *H. sapiens*, and *S. cerevisiae*.

Protein	Pfam domain	Identify (%)	Score
hsGLRX3	PF00462, Glutaredoxin, 2hits	35.4	448
	PF00085, Thioredoxin, 1 hit		
hsGLRX5	PF00462, Glutaredoxin, 1 hit	50.0	255
hsTXN	PF06201, PITH, 1 hit	29.5	164
	PF00085, Thioredoxin, 1 hit		
afCA14_001870	PF00462, Glutaredoxin, 1 hit	42.0	189
	PF00085, Thioredoxin, 1 hit		
af F9C07_2156032	PF00462, Glutaredoxin, 1 hit	41.6	181
	PF00085, Thioredoxin, 1 hit		
afAFLA70_126g002401	PF00462, Glutaredoxin, 1 hit	47.1	100
	PF01040, UbiA, 1 hit		
scGrx4	PF00462, Glutaredoxin, 1 hit	42.1	189
	PF00085, Thioredoxin, 1 hit		
scGrx3	PF00462, Glutaredoxin, 1 hit	43.4	206
	PF00085, Thioredoxin, 1 hit		

Note: Grx4 has two domains; PF00462, Glutaredoxin, 1 hit and PF00085, Thioredoxin, 1 hit.

#### 3.1.2 spFep1

**FIGURE 3:**
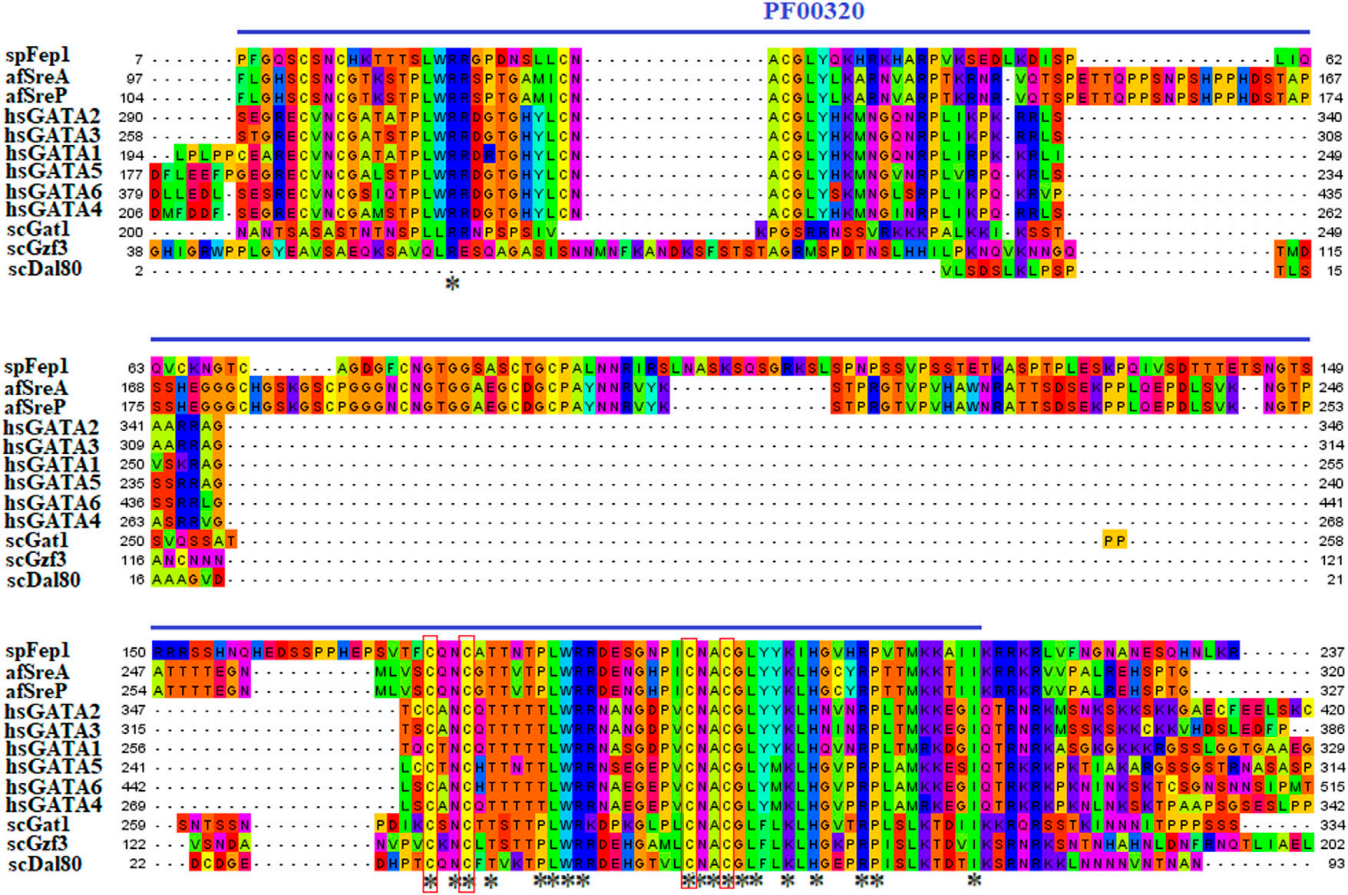
Multiple sequence alignment of sequences of the PF00320 domain in spFep1 and its sequence homologs in *A. flavus, S. cerevisiae,* and humans using the MEGA_X_10.1.8 software. The domain was identified by searching the SMART database. The four characteristic Cys residues are boxed while amino acid residues are highlighted according to the biochemical properties of the amino acid residues.

**FIGURE 4:**
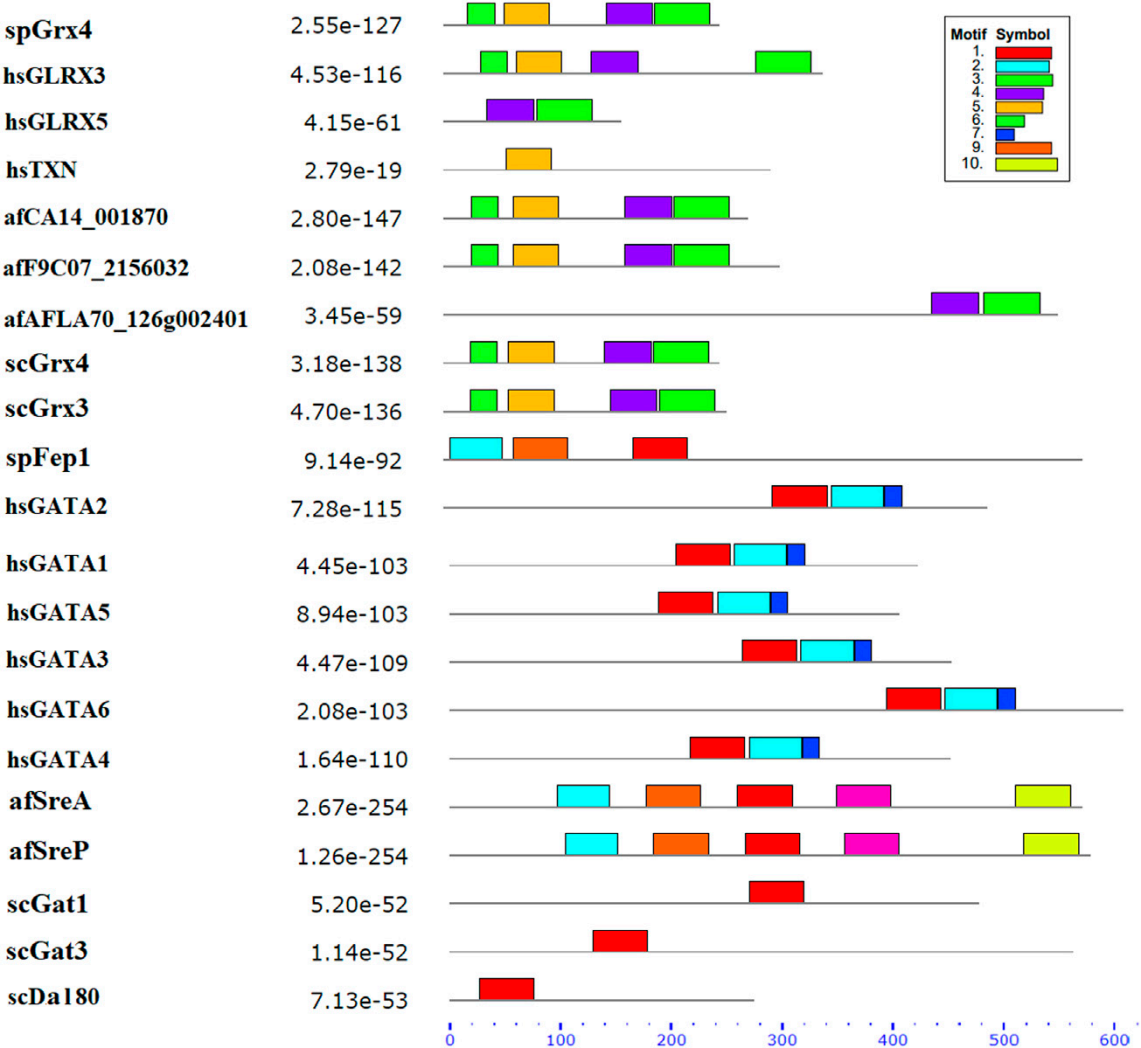
The conserved motif analysis of selected proteins in Figure 1 using the MEME Suite, where the motif composition of these proteins using 10 conserved motifs is represented by a unique color mentioned in the box on the top right.

**FIGURE 5:**
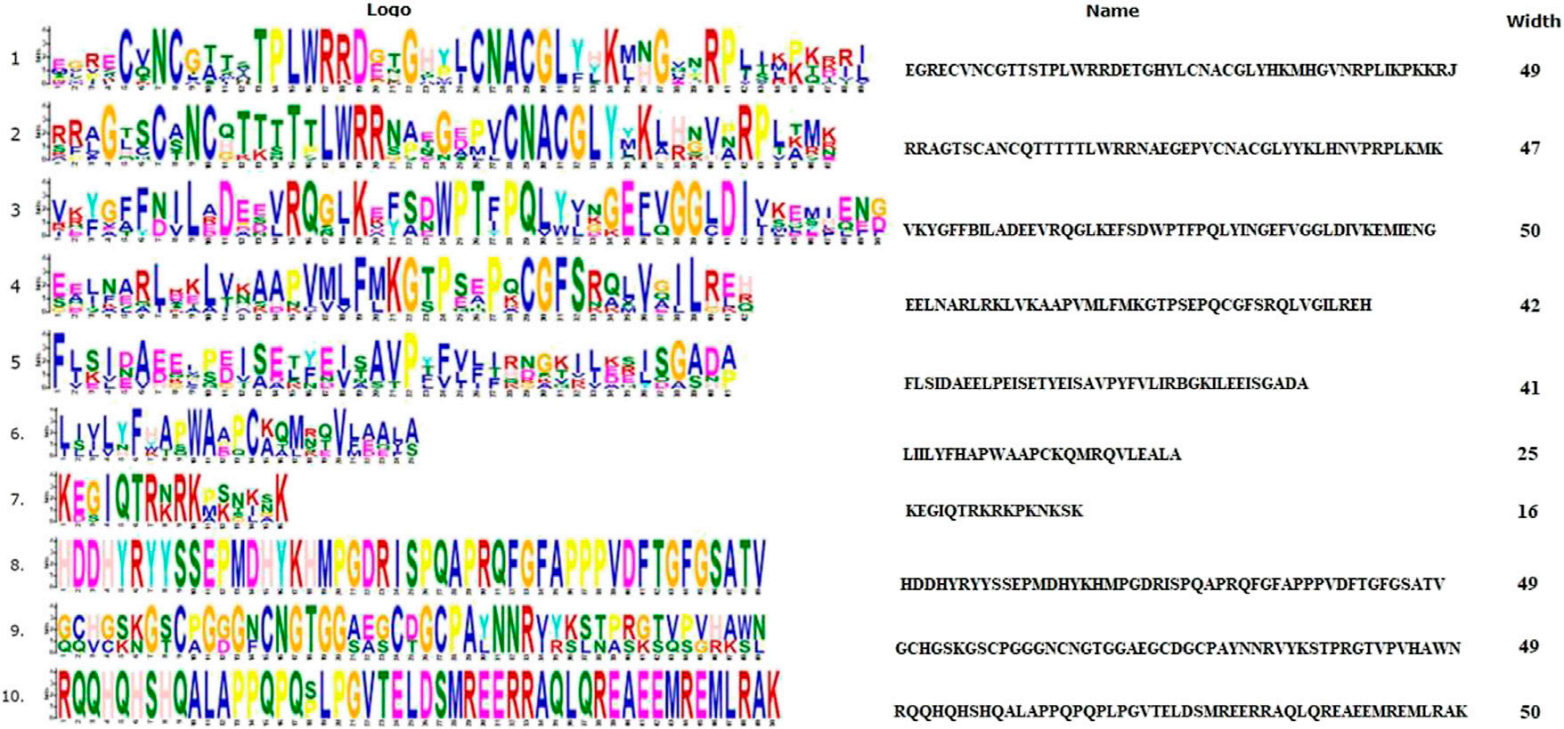
The 10 conserved motifs of selected proteins in Figure 1. The sequence logos was created by the MEME Suite.

Ten spFep1 homologs were chosen (Table 2): two from *S. cerevisiae* (scGat1 and scDal80), two from *A. flavus* (afSreA and afSreP), and six from humans (hsGATA1 to hsGATA6). According to the NCBI basic sequence blast results and the two major ortholog predictors (OrthoFinder and PHYLome) tools, scDal80 is most similar to spFep1, 60.9% identity (Table 2). To determine the evolutionary relationships between these Fep1 sequences, we constructed a phylogenetic tree of spFep1 and its sequence homologs using the maximum-likelihood method and a similarity score (Figure 1). Our study revealed that spFep1 is most similar to scDal80. Additionally, multiple sequence alignments of spFep1 and its sequence homologs in humans, *A. flavus* and *S. cerevisiae* were performed. This analysis identified four conserved Cys residues in all selected Fep1 PF00320 domains (Figure 3). According to the SMART database, our analysis revealed that spFep1 and its sequence homologs in human, *A. flavus* and *S. cerevisiae* all contain the GATA zinc finger (PF00320) domain, which is depicted in Figure 3. In addition, we discovered two highly conserved motifs in spFep1 (Figure 4) that are significant for iron transport systems because they contain residues involved in iron homeostasis (Figure 5).

**TABLE 2:** Sequence comparison of spFep1 to its sequence homologs in *A. flavus*, *H. sapiens*, and *S. cerevisiae*.

Protein	Pfam domain	Identify (%)	Score
hsGATA1	PF00320, GATA, 2 hits	42.9	209
hsGATA2	PF00320, GATA, 2 hits	36.2	215
hsGATA3	PF00320, GATA, 2 hits	43.5	196
hsGATA4	PF00320, GATA, 2 hits	55.0	186
	PF05349, GATA-N, 1 hit		
hsGATA5	PF00320, GATA, 2 hits	37.1	202
	PF05349, GATA-N, 1 hit		
hsGATA6	PF00320, GATA, 2 hits	48.1	194
	PF05349, GATA-N, 1 hit		
afSreA	PF00320, GATA, 2 hits	38.2	443
afSreP	PF00320, GATA, 2 hits	38.2	443
scGat1	PF08550, DUF1752, 1 hit	60.0	469
	PF00320, GATA, 1 hit		
scGzf3	PF00320, GATA, 1 hit	50.0	551
scDal80	PF00320, GATA, 1 hit	60.9	269

Note: Fep1 has one domain; PF00320, GATA, 2 hits.

#### 3.1.3 spPhp4

**FIGURE 6:**
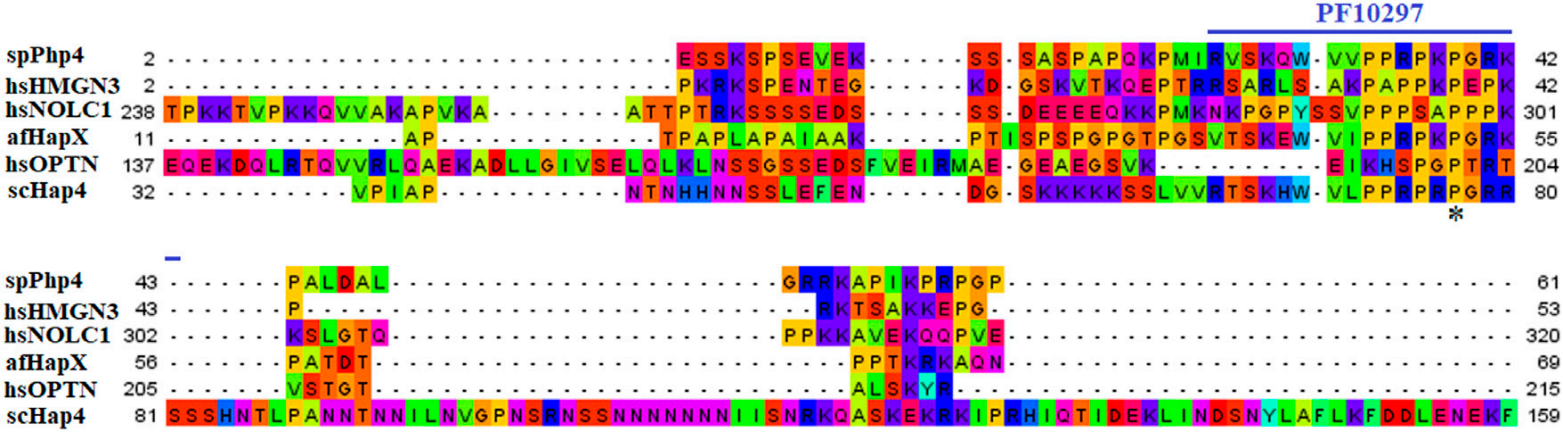
Multiple sequence alignment of sequences of the PF10297 domain in spPhp4 and its homologs in *A. flavus*, *S. cerevisiae* and humans. The domain was discovered through a search of the SMART database. They are highlighted according to the biochemical properties of the amino acid residues.

We chose 5 spPhp4 homologous: one from *S. cerevisiae* (scHap4), one from *A. flavus* (afHapX), and three from humans (hsHMGN3, hsNOLC1, and hsOPTN). The scHap4 is more similar to spPhp4 than others (Table 3). It shared the highest similarity with spFep1. The Php4 phylogenetic tree (Figure 1) demonstrates that spPhp4 is most related to scHap4. Furthermore, our multiple sequence alignment revealed the presence of two conserved Cys residues in spPhp4 and its homologs, despite the absence of these residues in its PF10297 domains (Figure 6). Based on the SMART database, our study demonstrated that *A. flavus, S. cerevisiae*, and spPhp4 all contain the Hap4_Hap_bind (PF10297) domain, as depicted in Figure 6. In addition, the MEME Suite identified a highly conserved motif in these Php4 (Figures 4, 5).

**TABLE 3:** Sequence comparison of spPhp4 to its sequence homologs in *A. flavus*, *H. sapiens*, and *S. cerevisiae*.

Protein	Pfam domain	Identify (%)	Score
hsHMGN3	PF01101, HMG14_17, 1 hit	26.9	84
hsNOLC1	PF05022, SRP40_C, 1 hit	23.4	82
hsOPTN	PF16516, CC2-LZ, 1 hit	31.5	81
	PF11577, NEMO, 1 hit		
afHapX	PF10297, Hap4_Hap_bind, 1 hit	37.7	130
scHap4	PF10297, Hap4_Hap_bind, 1 hit	39.2	147

Note: Php4 has one domain; PF10297, Hap4_Hap_bind, 1 hit.

### 3.2 3D structure and protein-protein interaction

**FIGURE 7:**
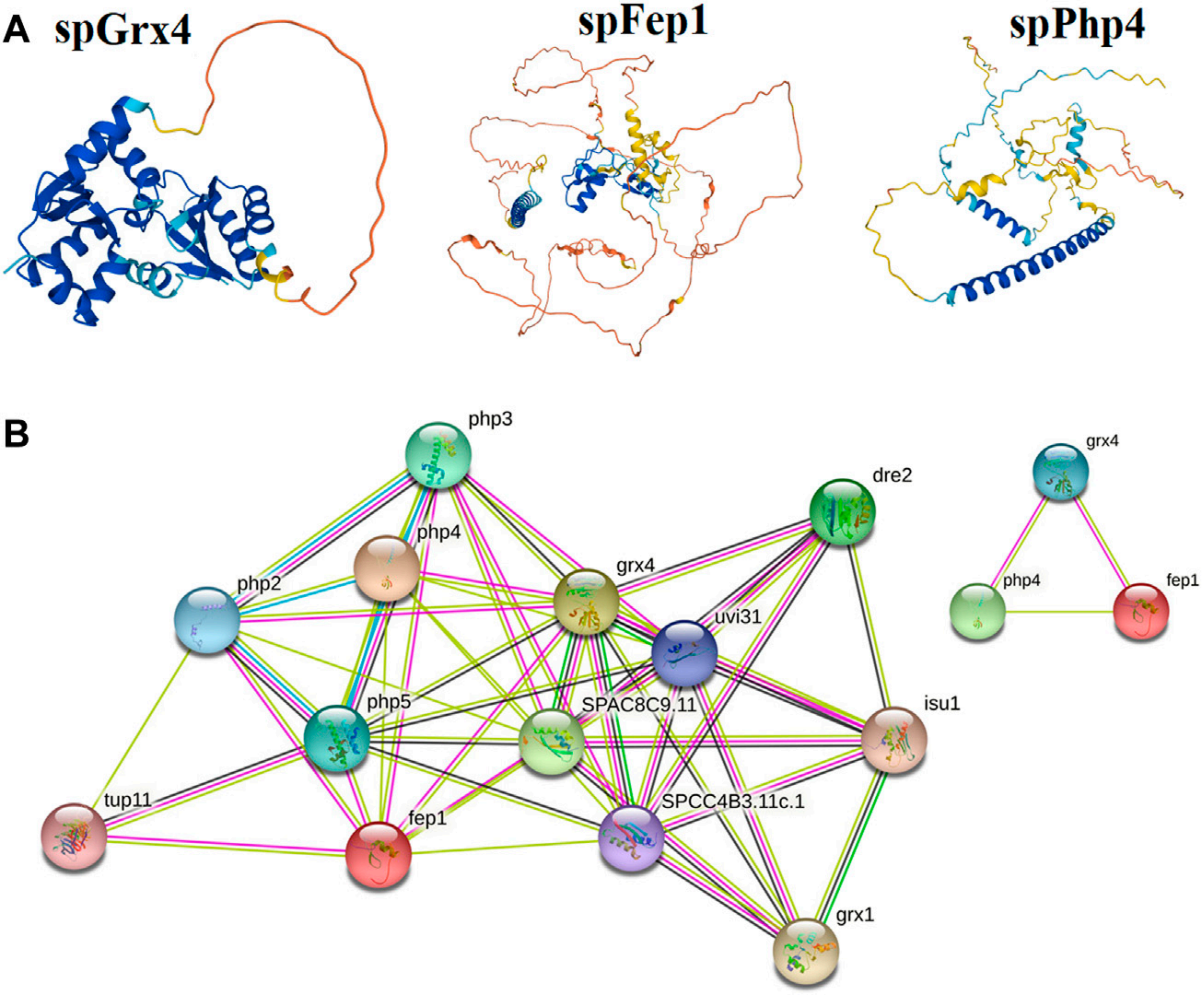
Protein analysis; **(A)** The predicted three-dimensional structures of spGrx4, spFep1, and spPhp4. **(B)** The Protein-protein interaction network building among spGrx4, spFep1, and spPhp4 was performed using the Phyre2 server in an intensive mode. Rainbow colors visualized protein models were visualized by rainbow colors in the direction from N to C terminus. Unknown 3D structures are represented by empty nodes, known or predicted 3D structures by filled nodes, query proteins, and the first shell of interactors by colored nodes, and the second shell of interactors by white nodes.

The spGrx4 protein contains 244 amino acids, with 28 negatively charged residues (Asp and Glu) and 19 positively charged residues (Arg and Lys). At its N-terminus, spGrx4 has an additional domain containing a WAAPCK motif, similar to the WCGPCK motif in thioredoxin’s active site (Figure 7). spFep1 contains 564 amino acids, 43 of which are positively charged (Arg and Lys) and 53 negatively charged (Asp and Glu). Moreover, two Cys2-Cys2 zinc fingers are present in the spFep1 protein (Figure 7). In addition, the Cys-X5-Cys-X8-Cys-X2-Cys motifs are positioned between the two zinc fingers (Figure 7). spPhp4 is composed of 295 amino acids, 38 (Asp and Glu) and 46 (Arg and Lys) of which are positively charged. Subsequently, using STRING to analyze protein-protein interactions, it was determined that spGrx4 interacts strongly with spPhp4 and spFep1. Furthermore, spGrx4, spPhp4, and spFep1 interact with spPhp2, spPhp3, and spPhp5, indicating that our three proteins play cooperative roles in iron homeostasis (Figure 7).

### 3.3 *spgrx4*, *spfep1,* and *spphp4* expression under different iron conditions

**FIGURE 8:**
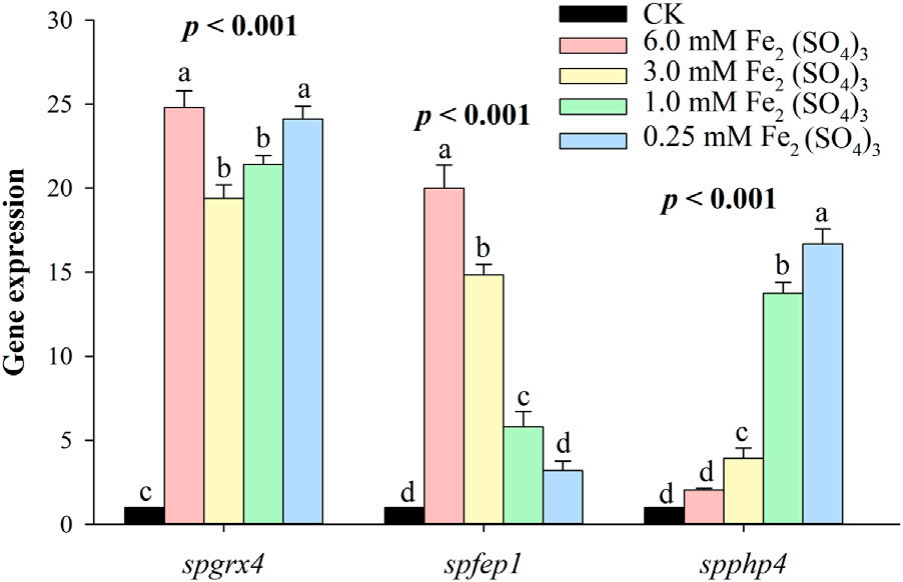
The qRT–PCR analysis of *spgrx4, spfep1* and *spphp4* at varying concentrations of ferrous. The β-actin gene served as the internal control. Error bars were used to depict the standard error of the three scientific repeats. The various letters represent statistically significant differences (*p* < 0.05) between the treatment and the control group.

At the highest level of iron, *spgrx4* expressed itself more than *spfep1*, while *spphp4* expressed itself the least. By decreasing the iron concentration, *spgrx4* continued to exhibit the highest expression level, while *fep1* expression decreased and *spphp4* expression increased (Figure 8). When iron levels are low, *S. pombe* cells inhibit the transcription of many genes that encode iron-utilizing proteins. spPhp4 represses iron utilization/storage genes by interacting with the CCAAT-binding Php complex composed of spPhp2, spPhp3, and spPhp5.

## 4 Discussion

Iron is a key element required for several fundamental organismal and cellular processes, including nucleic acid replication, mitochondrial respiration, oxygen transport, intermediary and xenobiotic metabolism, cell signaling, and host defense (Wang and Babitt, 2018). The property of iron as a transition metal to easily donate or accept electrons to contribute to oxidation reduction reactions is the basis on which these biological functions depend (Wang and Babitt, 2018). Cells must tightly regulate iron uptake to balance adequate and excessive iron levels (Katsarou and Pantopoulos, 2020). *S. pombe* uses two transcription factors, spFep1 under iron-abundant conditions and spPhp4 under iron-abundant conditions iron-deficient conditions, which are controlled by spGrx4 (Alkafeef et al., 2020). spFep1 and spPhp4 reciprocally regulate the expression of each iron-dependent genes, thereby coordinating their activities at the transcriptional level. Our analysis of the domain, protein structure, and gene expression patterns of the spGrx4, spFep1, and spPhp4 under different iron concentrations will help in understanding their roles in maintaining iron homeostasis. Our analysis showed that the human hsGLRX3 is most similar to the spGrx4, and that spGrx4 is strongly similar to scGrx4. All selected TRX (PF00085) domains in spGrx4 and its homologs contain a conserved cysteine (Cys) residue. In addition, our analysis revealed the presence of Cys^172^ in the GRX domain (PF00462). The highly conserved residues C172GFS, which are required for Grx4’s cellular functions, are located in the C-terminal GRX-like domain of the protein (Herrero and De La Torre-Ruiz, 2007). Furthermore, the strength of the interaction between spGrx4 and spPhp4 bound to iron depends on certain conserved Cys residues, as spGrx4 depends on Cys172 and spPhp4 depends on both Cys221 and Cys227 (Dlouhy et al., 2017). spGrx4 and its sequence homologs in human, *A. flavus* and *S. cerevisiae* all contain the thioredoxin (PF00085) and GRX (PF00462) domains (Figure 2), which contribute electrons to ribonucleotide reductase and are involved in thiol-redox and ribonucleotide reductase (Holmgren, 1989). In addition, yeast two-hybrid mapping reveals that the C-terminal region of spPhp4 physically interacts with spGrx4 *via* the C-terminal GRX-like and N-terminal TRX-like domains of spGrx4 (Vachon et al., 2012). Our analysis revealed that, scDal80 is the most similar to spFep1. However, scDal80 and spFep1 are not orthologs because they start serving different functions. scDal80 participates in nitrogen metabolism, whereas spFep1 participates in iron metabolism. scDal80 inhibits a number of independent inducer genes associated with nitrogen metabolism (Cunningham and Cooper, 1993). scDal80 negatively regulates a wide range of nitrogen-catabolic genes, and it encodes a protein with a zinc finger motif that bears extensive homology to the metazoan GATA-binding family of transcriptional activators (Cunningham and Cooper, 1993). It is worth to noting that scGzf3, which is a paralog of scDal80, inhibits nitrogen catabolic gene expression by competing with scGat1 for GATA site binding (Palavecino-Ruiz et al., 2017). These results are consistent with the notion that *S. cerevisiae* and *S. pombe* use different mechanisms to maintain iron homeostasis.

Our analysis identified four conserved Cys residues in all selected GATA zinc-finger domains (PF00320) in spFep1 and its homologs. A region containing four conserved Cys residues that regulate iron homeostasis in *S. pombe* is flanked by the DNA-binding motifs of these GATA-type transcription factors, which typically contain one or two zinc finger motifs (Attarian et al., 2018). We also found that spFep1 and its sequence homologs in human, *A. flavus,* and *S. cerevisiae* all contain the GATA zinc finger (PF00320) domain (Figure 3). This domain regulates iron homeostasis (Blaiseau et al., 2001). When cells are iron-replete, spFep1 binds to GATA sequences in these genes and inhibits their transcription, thereby downregulating iron transport systems (Dlouhy et al., 2017). Moreover, we observed two highly conserved motifs in spFep1 that are significant for iron transport systems because they contain residues involved in iron homeostasis.

We also showed that the scHap4 is more similar to spPhp4 and shared the highest degree of similarity with spFep1, and that the Cys-rich protein plays a significant iron-binding role. Comparable to a similar Cys-rich region with an iron-sensing function in HapX, the Php4 protein contains a Cys residue close to its carboxyl terminus (Hortschansky et al., 2007). By using genome-wide microarray analysis, Mercier et al. (2008) determined that spPhp4 is involved in the inhibition of 86 genes under iron-starvation conditions. Our study also demonstrated that spPhp4 and its sequence homologs in *A. flavus, S. cerevisiae*, and humans all contain the Hap4_Hap_bind (PF10297) domain. This domain is essential for scHap4 to form the scHap complex by allowing it to bind to scHap2, scHap3, and scHap5 (McNabb and Pinto, 2005). spGrx4 interacts with spPhp4 through its C terminus, which includes amino acid (aa) residues 152–254 (Mercier and Labbé, 2009). spPhp4 and scHap4 share a small amount of sequence homology overall. scHap4 contains a conserved domain, which is required for to link scHap4 to scHap2/scHap3/scHap5 (McNabb and Pinto, 2005). Furthermore, the MEME Suite identified a highly conserved motif in these spPhp4 (Figure 4). This motif is important for iron transport systems because it contains iron homeostasis-involved residues (Figure 5). Unlike spPhp4, scHap4 plays a role in controlling the fermentation/respiration switch (Lascaris et al., 2003). These results also support the notion that *S. cerevisiae* and *S. pombe* use different strategies to control iron homeostasis.

Our study also revealed that spGrx4 interacts strongly with spPhp4 and spFep1, and that spGrx4, spPhp4, and spFep1 interact with spPhp2, spPhp3, and spPhp5, indicating that our three proteins play cooperative roles in iron homeostasis (Gupta and Outten, 2020; Misslinger et al., 2021). spGrx3, spGrx4, and spGrx5 are three monothiol glutaredoxins found in *S. pombe* (Chung et al., 2005; Berndt et al., 2021)*.* They are primarily localized at the nuclear rim, throughout the cell (cytosol and nucleus), and in mitochondria, respectively (Chung et al., 2005; Mercier and Labbé, 2009). At the active site of spGrx3, spGrx4, and spGrx5, which is part of the GRX-like domain, one Cys residue is highly conserved (Chung et al., 2005; Berndt et al., 2021). Earlier research discovered that the spGrx3 and spGrx4 monothiol glutaredoxins contain the N-terminal TRX-like domain (Herrero and de la Torre-Ruiz, 2007). This domain is required for the nuclear localization of monothiol glutaredoxins containing the TRX domain (Molina et al., 2004). The spGrx4 protein is associated with oxidative, osmotic, nitrosative, and iron-dependent stress responses (Chung et al., 2005; Kim et al., 2005; Mercier and Labbé, 2009).

At the highest level of Fe, *spgrx4* expressed itself more than *spfep1*, while *spphp4* expressed itself the least. By decreasing the Fe concentration, *grx1* continued to exhibit the highest expression level, while *spfep1* expression decreased and spPhp4 expression increased (Figure 8). When iron levels are low, *S. pombe* cells inhibit the transcription of many genes that encode iron-utilizing proteins (Labbé et al., 2007; Liu et al., 2022). Interacting with the CCAAT-binding complex composed of spPhp2, spPhp3, and spPhp5, spPhp4 modulates transcription (Mercier et al., 2006). spPhp4 is inactivated under iron-rich conditions, allowing iron-dependent proteins to be translated (Mercier et al., 2006). *S. pombe* uses spFep1 and spPhp4 in iron-rich and iron-deficient conditions, respectively (Brault et al., 2015) to regulate the amount of iron in cells. Furthermore, *spfep1* and *spphp4* mutually regulate one another’s expression using iron-dependent mechanisms (Labbé et al., 2013). Moreover, spFep1, a GATA-type transcriptional inhibitor, primarily regulates iron acquisition related genes (Pelletier et al., 2002). Under iron-replete conditions, spFep1 binds to the GATA sequences in iron transport genes and inhibits their transcription, thereby downregulating the iron transport systems (Jbel et al., 2009; Jung et al., 2021; Miele et al., 2021). In *S. pombe,* spPhp2, spPhp3, and spPhp5 operate as heterotrimers to bind CCAAT cis-acting elements and activate gene expression (Mercier et al., 2006). This complex regulates the expression of genes encoding proteins involved in iron-dependent metabolic processes such as amino acid biosynthesis, iron-sulfur cluster assembly, the TCA cycle, mitochondrial respiration, and heme biosynthesis (Mercier et al., 2008). spPhp4 binds to the CCAAT-binding complex and transforms it from an activator to a suppressor in the absence of iron (Mercier et al., 2006). Therefore, Php4’s primary function is iron conservation, as depleted cellular iron inhibits iron-dependent protein-coding gene expression (Brault et al., 2016).

The activities of spFep1 and spPhp4 are also controlled post-translationally by the cytosolic CGFS glutaredoxin spGrx4 (Jbel et al., 2009; Mercier and Labbé, 2009; Encinar del Dedo et al., 2015). spGrx4 inhibits binding of spFep1 to the promoters of iron transport genes under iron-limiting conditions (Jbel et al., 2011; Encinar del Dedo et al., 2015). spGrx4 regulates interactions between spPhp4 and the spPhp2/3/5 complex (Mercier and Labbé, 2009). Under iron-depleted conditions, spPhp4 binds to the CCAAT-binding HAP complex (Php2/3/5) to suppress iron utilization/storage genes (Mercier and Labbé, 2009). However, when iron is present in high concentrations, spPhp4 dissociates from spPhp2/3/5 and is then re-localized to cytoplasm by spCrm1 (Mercier and Labbé, 2009). A yeast two-hybrid study revealed that spGrx4 physically interacts with spPhp4’s C-terminal region *via* its N- and C-termini TRX and GRX domains (Vachon et al., 2012). In contrast, iron-dependent binding to the TRX domain requires Cys172 at the conserved CGFS active site, whereas binding to the GRX domain is weak and constitutive. Cys172 is essential for spGrx4-dependent inhibition of spPhp4 in strains expressing C172 S/A spGrx4 mutants (Kim et al., 2011; Encinar del Dedo et al., 2015; Dlouhy et al., 2017). Cys221 and Cys227 in spPhp4 may be involved in the iron-mediated inactivation of spPhp4 by interacting with the GRX domain of spGrx4 (Dlouhy et al., 2017).

## 5 Conclusion

Here, we performed a bioinformatics analysis on *S. pombe* and their sequence homologs in *A. flavus*, *S. cerevisiae*, and *H. sapiens*, and an expression analysis with different iron concentrations to gain a deeper understanding of the critical roles of spGrx4, spPhp4, and spFep1 in iron homeostasis. Our analyses of Grx4 revealed a conserved cysteine residue in each TRX domain, and that human hsGLRX3 is most similar to spGrx4. Moreover, spFep1 is highly correlated with scDal80, whereas scHap4 is most similar to spFep1 and to spPhp4. Our analysis also showed that spGrx4 interacts strongly with spPhp4 and spFep1, and that spGrx4, spPhp4, and spFep1 interact with spPhp2, spPhp3, and spPhp5, indicating that our three proteins play cooperative roles in iron homeostasis. At the highest level of Fe, *spgrx4* had the highest expression, followed by *spfep1*, while *spphp4* had the lowest expression; a contrast occurred at the lowest level of Fe, where *spgrx4* expression remained constant. Our analysis improves our understanding of the structure and function of spGrx4, spFep1, and spPhp4.

## Data Availability

The original contributions presented in the study are included in the article/supplementary material, further inquiries can be directed to the corresponding author.
